# Postnatal Dynamics of Circulating Steroid Hormones in Mule and Equine Neonates

**DOI:** 10.3390/vetsci9110598

**Published:** 2022-10-28

**Authors:** Yatta Linhares Boakari, Erin Legacki, Maria Augusta Alonso, Ana Carolina Francisco dos Santos, Marcilio Nichi, Alan J. Conley, Claudia Barbosa Fernandes

**Affiliations:** 1 Department of Large Animal Clinical Sciences, College of Veterinary Medicine and Biomedical Sciences, Texas A&M University, College Station, TX 77843, USA; 2 Department of Population Health and Reproduction, School of Veterinary Medicine, University of California at Davis, Davis, CA 95616, USA; 3 Department of Animal Reproduction, School of Veterinary Medicine and Animal Science, University of São Paulo, Sao Paulo 05508-270, Brazil

**Keywords:** mass spectrometry, endocrinology, equids, foals

## Abstract

**Simple Summary:**

Neonatal pathologies are extremely important, such as neonatal maladaptation related to hormonal dysregulation in foals. As mules are becoming more popular, there is an increased demand for information on their health, including normal hormonal profiles of mule foals during the perinatal period and how it differs from equine foals. This study evaluated hormones (pregnanes, corticoids, and androgens) that are related to foal physiological development and maturation during the time neonates adapt to extra-uterine life; in mule and equine foals during their first 12 h after birth. Our findings suggest that there might be differences in the hormonal milieu between mule and horse foals, which might be related to the different placentation and uterine environment during hybrid pregnancies. Additionally, the pattern of hormones shows that there are derived from different tissues. These results can assist veterinarians with early diagnosis and intervention of neonatal mules with hormonal imbalances.

**Abstract:**

It is necessary to study hormonal patterns from mules to recognize alterations and neonatal maladaptation. Our objective was to evaluate concentrations of hormones in mule (*n* = 6) and equine foals (*n* = 6). Blood was collected at T0, 1, 6 and 12 h after birth. Hormone concentrations were evaluated using liquid chromatography tandem mass spectrometry. Effects of time, group and interactions and regression analysis were evaluated (*p* < 0.05). There was a cubic and quadratic decline in mule and equine foals, respectively, for 3β,20α-dihydroxy-DHP. Mule foals were born with lower circulating 3β,20α-dihydroxy-DHP concentrations, which might be related to progestogen concentrations in mares with a hybrid placenta. Corticosterone and cortisol concentrations remained unchanged for the first hour post-foaling then declined in mule and equine foals (*p* < 0.0001). Dehydroepiandrosterone was the main androgen present. There was a decrease in dihydrotestosterone at 12 h (*p* = 0.002). Differences in the temporal patterns of secretion within each steroid class, pregnanes, corticoids, and androgens, suggest they were derived from different tissue sources, presumptively the placenta, adrenals and gonads of the fetus/neonate, respectively. Mule and horse foals were born without evidence of testosterone secretion. For the first time, steroid hormone levels were measured in neonatal mules, and this will provide insight into neonatal physiology that differs from equine and allow us to gain an understanding of mules that have rarely been studied. Further studies are needed to elucidate the effects of hybrid pregnancies in the steroid endocrinology of neonates.

## 1. Introduction

Mules are hybrids, resulting from breeding between an asinine (*Equus asinus*, 2n: 62) sire and an equine (*Equus caballus*, 2n: 64) dam [1]. In comparison to horses, mules are considered “resistant” by virtue of their capacity to thrive on low quality diets and smaller water consumption [2]. They are also considered to be relatively easy animals to train and to manage, and are less reactive to outside stimuli when compared to horses [3]. Differences are even apparent from as early as foaling [4]. For instance, we observed that mule foals (*n* = 30) have a higher APGAR score when compared to equine neonates (*n* = 17) immediately after birth and 60 min after birth [4], which can be related to differences in progestogen clearance in the neonatal period. The popularity of mules has grown in recent years with their increasing use in competitive sporting events including roping, gaited competitions and trail rides [5,6]. Thus, the demand for healthy mule foals, with superior genetics has resulted in an increase in breeding them. Yet, little has been done to investigate their physiology.

The health of a pregnancy influences on neonatal viability. Adequate fetal development and homeostasis are maintained by the placental transfer of nutrients, metabolites and hormones between the maternal and fetal compartments [7]. Consequently, what appears in the fetus reflects components from the maternal circulation just as the fetus contributes to the maternal system, especially concerning steroid concentrations [7,8,9]. This likely remains true at foaling such that the endocrinological profile of neonatal mule foals probably reflects the profile existing immediately prior to delivery [8,9,10,11], at least initially. Moreover, the profile of steroid hormones in foals at birth can be an indication of neonatal health and viability. Notably, foals suffering from maladjustment syndrome have grossly abnormal concentrations of several steroid hormones, such as higher concentrations of dehydroepiandrosterone, progesterone and pregnenolone [12,13,14,15,16]. Additionally, in shock or suffering septicemia, foals have higher cortisol concentrations [17].

Despite the potential relevance as an index of vitality in foals at birth, few studies have investigated steroid hormone profiles in equine and donkey neonates [12,17,18] and, to the best of our knowledge, no studies have been done on mule foals. Thus, the objective of this study was to characterize and compare steroid profiles up to 12 h after birth in mule and equine foals, both males and females, using liquid chromatography tandem mass spectrometry.

## 2. Materials and Methods

### 2.1. Animals and Experimental Design

Samples for this study were collected during the foaling season of 2017/2018 in a breeding operation center located in Piracaia in São Paulo–Brazil (Latitude: 23°03′14″ S, Longitude: 46°21′29″ W). This study was approved by the Ethics Committee in Animal Experimentation (CEUA) from the School of Veterinary Medicine at University of Sao Paulo and is under the protocol number: 6001260715.

A total of 6 mule foals, 3 females and 3 males, from Mangalarga mares bred to a National breed donkey and 6 equine foals, 2 females and 4 males, from Mangalarga Paulista mares bred to a Mangalarga stallion were used. The mares were healthy and did not receive any medications during gestation. Mares ages at delivery was 13.54 ± 1.54 years (range 7 to 23 years). The inclusion criteria in this experiment were: neonates from normal foaling with no signs of clinical disease and parameters (behavior, mucous membrane color, capillary refill time, rectal temperature and heart and respiratory rates) within normal limits.

### 2.2. Sample Collection and Storage

Monitoring of mares was implemented when they neared 330 days post ovulation, or when parturition appeared to be imminent otherwise. Blood samples were collected from the neonates by jugular venipuncture using one vacutainer tube (BD Vacutainer^®^, Curitiba, Brazil), at four different time points: at birth (immediately after expulsion–T0), and at 1, 6 and 12 h after foaling (T1, T6 and T12, respectively). Immediately after the sample collection, samples were centrifuged (151× *g* for 10 min) to separate the serum. Subsequently, the samples were aliquoted and stored in cryovials at −20 °C until assays were conducted.

### 2.3. Sample Analysis

Mass spectrometry was performed for the following hormones: pregnenolone, progesterone, 17αOH-progesterone (17-OHP), 5α-dihydroprogesterone (DHP), allopregnanolone, 20α-hydroxy-DHP (20α-DHP), 3β,20α-dihydroxy-DHP (3β,20α-DHP), dehydroepiandrosterone (DHEA), androstenedione, 19-norandrostenedione (19-norA4), testosterone, dihydrotestosterone (DHT), estrone, corticosterone and cortisol. Sample analyses were performed at the Clinical Endocrinology Laboratory at the School of Veterinary Medicine, University of California at Davis (Davis, CA, USA) as previously described [19] using 200 µL of serum.

### 2.4. Statistical Analyses

The data were analyzed using the SAS program 9.3 (SAS Institute Inc., Cary, NC. USA, 2018). The assumptions of normality of residues and homogeneity of variances were tested and transformation was applied to 3β,20α-DHP, cortisol, progesterone and pregnenolone. Effect of group, time and interaction were analyzed with the General Linear Mixed Model (PROC MIXED). Group and time effect were considered separately when group-by-time interaction was not significant using a Tukey’s Studentized Range (HSD) test. The regression analyses were performed using the Guided Data Analyses from SAS. The significance was set at *p* ≤ 0.05. Data are shown as mean and standard error of the mean.

## 3. Results

The mean gestational length for the mares used in this study was 345.8 days (range of 335–362 days). There was no statistical difference (*p* > 0.05) between gestational length of mares pregnant with mule or equine foals. At the time of birth, the same steroids were detected in mule and horse foals including pregnenolone (range of 1000–3000 ng/mL), and in order of decreasing concentration relative to one another, 3β,20α-DHP, cortisol, DHEA, progesterone, corticosterone and androstenedione, as well as trace amounts of DHT (<1 ng/mL, Table 1). Several steroids including 17αOH-progesterone, DHP, allopregnanolone, 20α-DHP, 19-norandrostenedione, testosterone and estrone were not detected. There were too few foals of each sex for a truly valid analysis but there was no statistical difference (*p* > 0.05) in any of the steroids measured between male and female mule or horse foals, thus data for each group were combined for further analysis.

**Figure 1:**
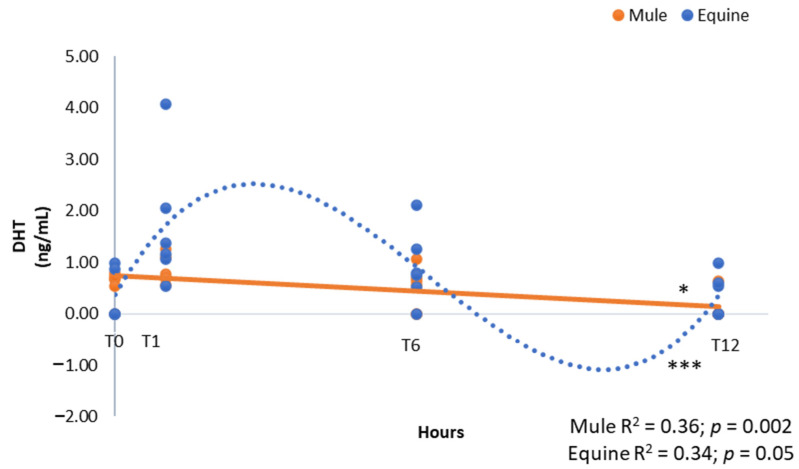
Androgen concentrations of dihydrotestosterone (DHT, ng/mL) in neonatal mule (*n* = 6, solid line) and equine (*n* = 6, dashed line) foals at birth (immediately after expulsion–T0), and at 1 (T1), 6 (T6) and 12 h after birth (T12). Results represent the mean. * Linear and *** cubic effects are shown with R2 and *p*-values.

No differences were obtained between groups and among the time points for androstenedione and DHEA (Table 1). DHEA was the predominant androgen present in mule and equine foals (range of 0.00–63.05 ng/mL). There was a linear decline of DHT in mule foals (Figure 1, R^2^ = 0.36, *p* = 0.002) and a cubic decline in equine foals (Figure 1, R^2^ = 0.34, *p* = 0.05).

Group effect and time effect were considered when group-by-time interaction was not significant. *p*-values and interaction between group and time are shown. Significance was set at *p* ≤ 0.05.

**Figure 2:**
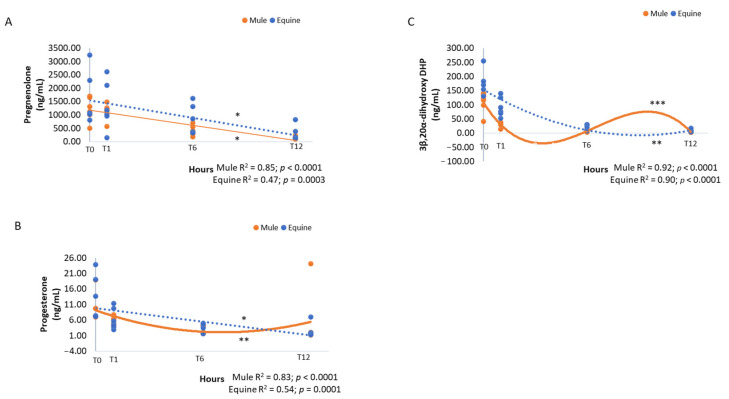
Pregnane concentrations of pregnenolone (**A**), progesterone (**B**) and 3β,20α-dihydroxy DHP (**C**) (ng/mL) in neonatal mule (*n* = 6, solid lines) and equine (*n* = 6, dashed lines) foals at birth (immediately after expulsion–T0), and at 1 (T1), 6 (T6) and 12 h after birth (T12). Results represent the mean. * Linear, ** quadratic and *** cubic effects are shown with R^2^ and *p*-values.

The concentrations of pregnanes exhibited a similar general pattern of a steady, significant decline with time after delivery (Table 2, *p* < 0.0001). While pregnenolone had a linear decline in mule and equine foals (Figure 2, R^2^ = 0.85, *p* < 0.0001 and R^2^ = 0.47, *p* = 0.0003, respectively), progesterone exhibited a decline that was quadratic in mules (Figure 2, R^2^ = 0.83, *p* < 0.0001) and linear in equine foals (Figure 2, R^2^ = 0.54, *p* = 0.0001), over time. Mule and equine foals exhibited a cubic (Figure 2, R^2^ = 0.92, *p* < 0.0001) and quadratic (Figure 2, R^2^ = 0.90, *p* < 0.0001) decline, respectively in 3β,20α-DHP, a 5α-reduced metabolite of progesterone, with lower concentrations in mule foals (Table 1, *p* = 0.003).

**Figure 3:**
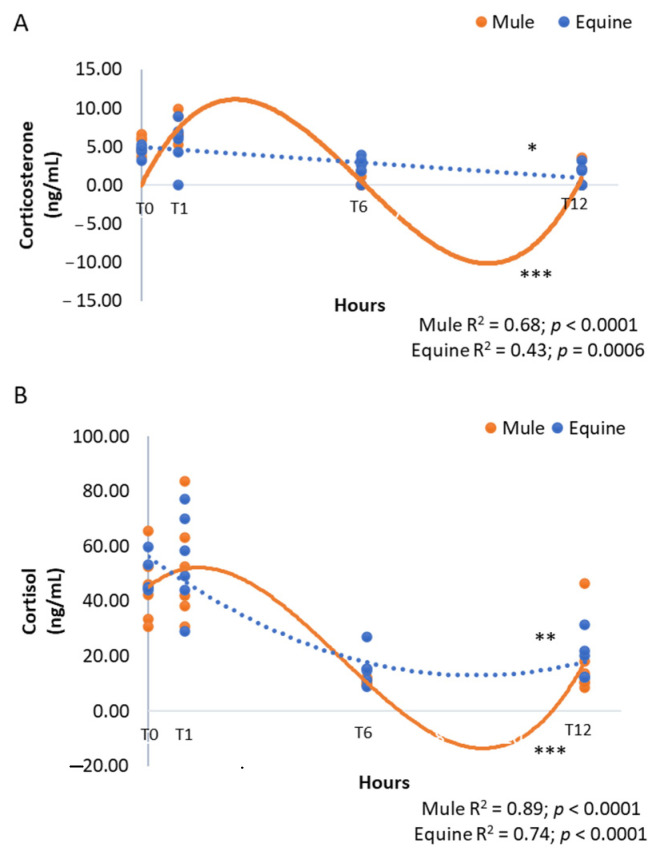
Corticoid concentrations of corticosterone (**A**) and cortisol (**B**) (ng/mL) in neonatal mule (*n* = 6, solid lines) and equine (*n* = 6, dashed lines) foals at birth (immediately after expulsion–T0), and at 1 (T1), 6 (T6) and 12 h after birth (T12). Results represent the mean. * Linear, ** quadratic and *** cubic effects are shown with R^2^ and *p*-values.

The disappearance with time of pregnenolone, progesterone, and 3β,20α-DHP contrasted with sustained corticosterone (averaging 4–5 ng/mL at foaling) and cortisol (averaging 40–50 ng/mL) concentrations (Table 2). Corticosterone and cortisol remained unchanged for the first hour post-foaling then declined rapidly to relatively low levels in samples taken at the 6 and 12 h (*p* < 0.0001, Table 2). The decline was best described as cubic for mule foals (Figure 3, R^2^ = 0.68, *p* < 0.0001) and linear for equine foals (Figure 3, R^2^ = 0.43, *p* = 0.0006) for corticosterone and cubic for mule foals (Figure 3, R^2^ = 0.89, *p* < 0.0001) and quadratic for equine foals (Figure 3, R^2^ = 0.74, *p* < 0.0001) for cortisol.

## 4. Discussion

The immediate post-partum period is a physiological challenge for neonates as they adapt to extra-uterine life, responding to metabolic, respiratory and other physiological demands [20], some of which have an endocrine signature. Both equine and mule foals at birth have substantial concentrations of several steroid hormones, among them pregnane metabolites of progesterone and its substrate, pregnenolone, being the most abundant. The high concentrations of 3β,20α-DHP and pregnenolone present at delivery likely reflect the predominance of 3β,20α-DHP in the maternal circulation pre-partum, [13,19,21] and the high pregnenolone concentrations found in the fetus [22]. These pregnanes are cleared effectively within a few hours of delivery in normal foals [12], as confirmed here with concentrations decreasing during the first 12 h after birth for both horse and mule foals. However, in dysmature (full-term with premature clinical signs) or maladjusted (exhibit weakness, incoordination, convulsions, etc.) foals, pregnane concentrations can remain elevated, especially in foals that do not recover [12,14]. In addition to pregnanes, androgens including DHEA and androstenedione have been detected in neonatal foals and have been assumed to be of adrenal origin also [14]. However, other tissues like the gonads are capable of contributing to steroid concentrations in neonatal foals [9], as can the placenta pre-partum [9,23]. Just as importantly, circulating steroid concentrations are determined as much by clearance as they are by production.

Clues to the origin of circulating steroids in neonatal foals might be seen by a comparison of changes in concentration immediately after foaling. The data presented here confirm earlier reports in terms of the major pregnanes detected but extend them in examining corticoids as well as androgens in not only neonatal horses but also mules. Three different patterns of secretion were evident defining each class of steroid. This suggests that steroids within each class, pregnanes, corticoids and androgens, arise from the same tissue source and are cleared, or not, in a similar manner in the immediate neonatal period after delivery. Specifically, the monophasic decline in 3β,20α-DHP, pregnenolone and progesterone are consistent with clearance in the absence of ongoing synthesis, presumably by the placenta and/or endometrium pre-partum. Delivery at birth precludes both contributions from, and clearance of placentally derived steroids. In contrast, both cortisol and cortisone in neonatal horses and mules declined between 1-6 h of life to concentrations that were then maintained to 12 h. This is consistent with an activated adrenal gland with elevated corticoid secretion that was sustained post-partum for at least an hour but then stabilized at lower concentrations as foals acclimated. In contrast to both pregnanes and corticoids, there was little evidence of any consistent change in androgen concentrations over the period investigated. This is perhaps indicative of secretion from the fetal gonads not the fetal adrenal, though this has been implied in past studies on maladjusted foals that exhibit significantly elevated DHEA over their normal contemporaries [14]. The results observed here suggest that elevations in maladjusted foals might be attributable to reduced steroid clearance resulting from compromised renal or hepatic function. Further studies are needed to confirm or refute this possibility.

Despite prior interest in endocrine studies on neonatal horses and donkeys [12,13,18,24,25,26], no similar data have been reported in the immediate neonatal period in mule foals. As hybrids of horses and donkeys, mules are known to exhibit anatomical and physiological traits that differ from their parental species [27,28,29,30]. This includes a commonly perceived hardiness, as indicated perhaps by a superior resistance to heat stress [31]. Conceivably, physiological differences could emerge in utero during fetal development but very few studies have compared physical or physiological traits between mules and their parental stock at any stage of development [27,29]. Mule foals were born with lower circulating 3β,20α-DHP concentrations, which presumably reflect differences in concentrations circulating in mares carrying a hybrid placenta. To date, it has not been possible to investigate steroid profiles throughout gestation in mares carrying hybrid pregnancies. However, healthy pregnant jennies during the peripartal period showed differences in the progesterone pattern than what has been reported in the literature for mares [32]. No differences were observed in the present study for horse and mule foals corticoid profiles over time and results with higher cortisol concentrations at birth are similar to what has been reported previously for equine and donkey foals [18,26,33]. If differences in adrenocortical function exist between horse and mule foals in the neonatal period, they are small and likely not significant, from a physiological perspective.

There was only a small number of horse or mule foals of either sex, therefore male and female data from each group were combined for statistical analysis. However, it is still notable that no testosterone was detected in any of the male equine or mule foals. The limit of quantification and limit of detection (LOD) 235-for testosterone is 0.10 and 0.05 ng/mL, respectively, as reported previously [21]. Prior cross-sectional studies have reportedly detected testosterone even in fillies on the day of foaling in samples analyzed by immuno-assay [10,11]. The present LC-MS/MS results suggest otherwise. As stated, testosterone was not detected in our analysis of samples from neonatal foals of either sex or species. The principle androgens detected were, DHEA (15–25 ng/mL), DHT (0.05–0.86 ng/mL) and androstenedione (0.16–0.63 ng/mL). Despite small numbers of foals, if differences in testosterone exist between male and female neonates at birth, they are not obvious based on our analysis. It may be that testosterone is indeed present but at concentrations so low it is not detectable by this method of analysis. It is equally likely that the cross-reactivity of primary antisera are detecting other androgens instead, DHEA perhaps, given the concentrations measured and reported here. If so, residual interstitial tissue in ovaries and testes would be the most likely source. Neonatal testes exhibit a brief period of active testosterone secretion at or soon after birth in many species [34,35,36,37] and this is important in de-feminizing sexual behavior. A recent study reported that colts had measurable concentrations of testosterone from 0.2–0.5 ng/mL during their first year of life [38] but measurements were made using a commercial enzyme-linked immunoassay for testosterone that had cross-reactivities with other androgens, namely DHEA, androstenedione and DHT, 0.3–12.9%. We detected all of these androgens in both male and female neonates by LC-MS/MS. Testosterone concentrations in filly foals were apparently not evaluated or were not reported in the previous publication [38]. Similarly, 0.62 ng/mL of testosterone in newborn colts [11] and 0.75–0.95 ng/mL of testosterone in newborn filly foals [10] were reported. Both of these studies used an immunoassay without reporting the cross-reactivities of the primary antisera. Regardless, consistent with our data then, these researchers did not find higher testosterone in newborn colts than fillies. This is important because androgens from residual fetal gonadal interstitial tissue would be expected to be similar in colts and fillies and would not be expected to include testosterone. To this point, no quantifiable testosterone was found in gonads from equine fetuses at any stage of gestation from 4–10 months of pregnancy [9]. The interstitial tissues in equine fetal gonads are also known to secrete B-ring unsaturated androgens [39,40], the cross-reactivities of which in the EIAs used previously [10,25,38] are unknown. In short, immunoassays for testosterone could detect a variety of androgens in neonatal foals with residual gonadal interstitial tissue when testosterone is in fact less than 0.05 ng/mL, the LOD for our LC-MS/MS method. In our opinion, this is not only possible but likely and the detection of testosterone in neonatal foals by immunoassays is a case of “mistaken identity”. Our data suggests that male horses (and mules) do not experience a neonatal increase in testosterone, and surprisingly little is known about what influences the development of sexual behavior in stallions. In any case, testicular testosterone secretion in the post-natal period is not initiated until puberty in colts [25,38].

One limitation from this study was that the concentrations of pregnanes, corticoids, and androgens were not assessed in the pregnant mares to evaluate if there were differences between mares carrying mule or equine foals. This was not the objective of this study, however the differences in the hormonal profiles of mule and equine neonates might be better explained if the hormonal profile of the dams is investigated, thus, additional studies are needed to compare the hormonal profile of mares pregnant with mule or equine foals.

## 5. Conclusions

The current study documents and compares the steroid profiles of neonatal horse and mule foals for the first time, demonstrating that it differs in a couple of important ways. Horse and mule foals are born with a high concentration of 3β,20α-DHP (being lower in mule foals), a major metabolite of progesterone in pre-partum mares, and an apparent lack of testosterone secretion by the neonatal testis. Further investigation into the steroid endocrinology of hybrid pregnancies and mules themselves may help to further elucidate the influence of maternal and paternal genomes [41,42] on placental and fetal development.

## Figures and Tables

**Table 1:** Mean (±standard error) of steroid hormones from mule (*n* = 6) and equine foals (*n* = 6) collected during the first 12 h after birth.

Hormones (ng/mL)	Group		*p*-Value		
	Mule	Equine	Group	Time	Group × Time
**Androstenedione**	0.16 ± 0.07	0.63 ± 0.18	0.18	0.45	0.98
**3β,20α-dihydroxy DHP**	37.58 ± 9.56	69.86 ± 15.13	**0.003**	**<0.0001**	0.11
**Corticosterone**	3.48 ± 0.65	3.28 ± 0.51	0.77	**<0.0001**	**0.05**
**Cortisol**	31.38 ± 4.47	33.88 ± 4.33	0.28	**<0.0001**	0.73
**Dehydroepiandrosterone**	14.98 ± 3.11	27.45 ± 3.15	0.12	0.06	0.35
**Dihydrotestosterone**	0.50 ± 0.08	0.86 ± 0.20	0.16	**0.0005**	0.17
**Progesterone**	6.02 ± 1.14	6.23 ± 1.23	0.82	**<0.0001**	0.66
**Pregnenolone**	726.19 ± 111.35	1002.55 ± 180.63	0.36	**<0.0001**	0.17

The bold on the tables indicates the *p*-values that are smaller than 0.05.

**Table 2:** Mean (±standard error) of steroid hormones from mule (*n* = 6) and equine groups (*n* = 6) at birth (immediately after expulsion–T0), and at 1, 6 and 12 h after foaling showing time effect.

Hormones (ng/mL)	TIME				*p*-Value
	T0	T1	T6	T12	
**3β,20α-dihydroxy DHP**	140.59 ± 16.12 ^A^	61.40 ± 11.54 ^B^	12.91 ± 2.64 ^C^	5.88 ± 1.19 ^D^	**<0.0001**
**Corticosterone**	4.87 ± 0.31 ^A^	6.35 ± 0.74 ^A^	1.37 ± 0.41 ^B^	1.06 ± 0.40 ^B^	**<0.0001**
**Cortisol**	47.11 ± 3.12 ^A^	53.24 ± 5.12 ^A^	13.07 ± 1.43 ^B^	18.22 ± 3.18 ^B^	**<0.0001**
**Dihydrotestosterone**	0.48 ± 0.12 ^B^	1.28 ± 0.28 ^A^	0.70 ± 0.17 ^A,B^	0.23 ± 0.10 ^B^	**0.002**
**Progesterone**	11.90 ± 1.82 ^A^	6.28 ± 0.72 ^A^	2.88 ± 0.38 ^B^	3.93 ± 1.89 ^B^	**<0.0001**
**Pregnenolone**	1433.18 ± 233.87 ^A^	1238.09 ± 184.18 ^A^	605.20 ± 129.73 ^B^	216.90 ± 59.26 ^C^	**<0.0001**

^A,B,C,D^: Different letters on each row indicate statistical difference (*p* ≤ 0.05) between treatments. *p*-values are shown. Significance was set at *p* ≤ 0.05. The bold on the tables indicates the *p*-values that are smaller than 0.05.

## Data Availability

The data presented in this study are available on request from the corresponding author.
